# Unmasking Probable Bickerstaff Brainstem Encephalitis in the Absence of Radiological and Cerebrospinal Fluid Clues

**DOI:** 10.7759/cureus.109352

**Published:** 2026-05-21

**Authors:** Sachin Paul, Devasena Srinivasan, Viswanathan Pandurangan

**Affiliations:** 1 General Medicine, Sri Ramachandra Institute of Higher Education and Research, Chennai, IND

**Keywords:** autoimmune, brainstem, demyelination, encephalitis, seizures

## Abstract

Bickerstaff brainstem encephalitis (BBE) is a rare immune-mediated neurological disorder characterized by a wide spectrum of clinical presentations, often following a preceding infection. Although supportive findings on neuroimaging, cerebrospinal fluid (CSF) analysis, and antiganglioside antibodies may aid diagnosis, the condition remains largely clinical, particularly in atypical cases. We report the case of a 60-year-old man who presented with acute onset of fever and altered sensorium, rapidly progressing to severe quadriparesis and respiratory failure requiring mechanical ventilation. Neuroimaging and CSF analysis were unremarkable, with no evidence of albuminocytological dissociation. Nerve conduction studies revealed severe sensorimotor neuropathy involving all four limbs. In view of poor response to empirical antimicrobial therapy and after exclusion of infectious etiologies, we suspected an autoimmune process and initiated the patient on intravenous immunoglobulin (IVIG). We observed significant clinical improvement from the second dose onward, with progressive recovery of sensorium, motor power, and respiratory function. We successfully weaned the patient off ventilatory support, decannulated, and discharged him without residual neurological deficits. This case highlights the diagnostic challenge that atypical presentations of Bickerstaff encephalitis pose in the absence of classical radiological and CSF findings. It underscores the importance of maintaining a high index of clinical suspicion and initiating timely immunotherapy, even when conventional diagnostic markers are lacking.

## Introduction

Bickerstaff brainstem encephalitis (BBE) is a rare, immune-mediated demyelinating disorder of the central nervous system (CNS) first described by Edwin Bickerstaff in 1951 and further characterised in 1957. Drowsiness was identified as a consistent clinical feature in older case reports. BBE is characterised by brainstem dysfunction. Its typical presentation includes the clinical triad of ophthalmoplegia, ataxia, and altered consciousness. The disease often follows a preceding infection or, less commonly, post vaccination. Its estimated annual incidence is approximately 0.78 cases per 100,000 individuals [1,2].

BBE is part of the spectrum of antiganglioside antibody-mediated disorders, closely related to Miller-Fisher syndrome and Guillain-Barré syndrome (GBS). Its pathophysiology likely involves molecular mimicry, in which infectious agents trigger antiganglioside antibodies, particularly anti-GQ1b, that cross-react with the peripheral and CNSs. Anti-GQ1b antibodies are detectable in about two-thirds of patients, supporting their pathogenic role.

Neuroimaging and cerebrospinal fluid (CSF) analysis may offer supportive evidence, but such findings are inconsistent. MRI abnormalities are reported only in a minority of cases, while CSF results may be normal, show mild pleocytosis, or display albuminocytological dissociation [3,4]. Some patients may also show electroencephalographic abnormalities. Therefore, diagnosis of BBE is primarily clinical, supported by ancillary tests.

It is important to differentiate BBE from other anti-ganglioside antibodies. Detailed observation of the wider clinical picture, including ataxia, ophthalmoplegia, altered consciousness, and the pattern of paresis, aids in differentiation.

Given the variability in presentation and the absence of definitive diagnostic markers in a significant proportion of patients, BBE can pose a considerable diagnostic challenge. The diagnostic CSF markers might not be within reach in many developing nations. Early recognition is crucial because the timely initiation of immunotherapy is associated with favourable neurological outcomes. In this report, we describe a case of probable BBE in which classical radiological and CSF findings were absent, highlighting the importance of clinical suspicion in establishing the diagnosis.

## Case presentation

A 60-year-old South Asian man with Alzheimer’s disease, coronary artery disease (post-coronary artery bypass graft surgery), type II diabetes mellitus, and systemic hypertension on regular medication presented to the ER with one day of fever and altered sensorium. He had a 15-year history of chronic alcohol use (last binge 10 days prior) and tobacco sniffing. There was no history of seizures, vomiting, diarrhea, head or neck pain, dysuria, cough, head injury, trauma, ear pain or discharge, skin rashes, recent travel, or vaccination. There were no signs of recent upper respiratory or gastrointestinal infection.

On examination, the patient was drowsy with a pulse of 150/min, blood pressure of 80/60 mmHg, and oxygen saturation of 96% on room air. He was intubated for a low sensorium (Glasgow Coma Scale (GCS) E1 V2 M1). There was no neck stiffness on examination. Kernig’s and Brudzinski’s signs were negative. No rash, eschar, or lymphadenopathy was observed. Higher mental function and voluntary gaze could not be assessed due to poor sensorium. The oculocephalic reflex was absent, indicating brainstem oculomotor pathway dysfunction suggestive of ophthalmoplegia. Motor power was 0/5 in all limbs, with decreased tone and hyporeflexia; plantar reflexes were absent bilaterally. Sensory and cerebellar examination was not possible in view of low sensorium. Other systemic examinations were unremarkable. Laboratory tests showed polymorphonuclear leukocytosis, acute kidney injury, and high anion gap metabolic acidosis (Table 1). Arterial blood gas confirmed diabetic ketoacidosis, and insulin infusion was titrated according to blood sugars. The tropical fever panel, which tested for dengue, scrub typhus, malaria, and leptospirosis, was negative. Urine drug screening showed no evidence of substance abuse. Given fever, altered sensorium, and seizures, an acute CNS infection was suspected, and empirical antibiotics (ceftriaxone 2 g IV twice daily and acyclovir 750 mg IV three times daily) were started after lumbar puncture. CSF analysis showed no pleocytosis, normal protein, and elevated glucose, and was negative for autoimmune and infectious etiologies (Table 2). Brain CT and MRI were normal; spine MRI showed only degenerative changes. The patient had two generalized tonic-clonic seizures; the EEG done showed sharp wave discharge suggestive of generalized epileptiform activities-ictal activity. Antiepileptics were added, and no further seizure episodes were noted. A repeat EEG done on day 4 showed predominant delta activity with no epileptiform activity, suggestive of diffuse cerebral dysfunction. Nerve conduction studies were diagnostic, revealing absent compound muscle action potentials (CMAPs) in the bilateral upper and lower limbs, with non-recordable latencies, amplitudes, and conduction velocities. F waves were absent, and sensory nerve action potentials (SNAPs) were also non-recordable. Overall, the findings were suggestive of severe sensorimotor neuropathy involving all four limbs. Tracheostomy was performed for prolonged ventilation. Given persistent low sensorium, minimal response to antibiotics, prolonged ventilation, normal neuroimaging, and severe sensorimotor neuropathy, the differential diagnoses considered were: 1. Bickerstaff encephalitis; 2. atypical variant of GBS.

**Table 1:** Basic investigations

TEST	VALUES	NORMAL RANGE
HEMOGRAM		
Hemoglobin (g/dl)	14.5	13-17
Total counts (cells/mm3)	14,940	4000-11,000
Differentials		
Neutrophils (%)	80	45-70
Lymphocytes (%)	14.7	25-40
Eosinophils (%)	0.5	00- 06
Platelets (lakhs/mm^3^)	2.06	1.5-4.5
RENAL FUNCTION		
Blood urea nitrogen (mg/dl)	30	Jun-20
Creatinine (mg/dl)	1.5	0.7-1.2
Sodium (mmol/l)	147	135-145
Potassium (mmol/l)	5.2	3.5-5.1
Chloride (mmol/l)	114	98-107
Bicarbonate (mmol/l)	9	22-29
LIVER FUNCTION		
Total bilirubin (mg/dl)	1.15	<1.2
Direct bilirubin (mg/dl)	0.58	<0.3
Aspartate aminotransferase (IU/L)	46	<40
Alanine aminotransferase (IU/L)	19	<40
Albumin (g/dl)	3.9	3.5-5.2
Alkaline phosphatase (IU/L)	89	32-120

**Table 2:** Cerebrospinal fluid analysis

TEST	VALUE	NORMAL RANGE
APPEARANCE		
Opening pressure (cm of H_2_O)	10	6 to 20
Colour	clear	
CELL COUNT AND DIFFERENTIALS		
WBC (Cells/mm^3^)	1	0-5
Polymorph (%)	0	-
Monocyte (%)	100	-
CHEMICAL ANALYSIS		
Glucose (mg/dl)	150	40-70
Protein (mg/dl)	41.1	15-45
Chloride (mmol/l)	139	110-125
Adenosine deaminase (U/L)	6	-
Lactate (mg/dl)	19.54	Oct-22
MICROBIOLOGICAL ANALYSIS		
Gram stain	Negative	
Bacterial culture	Negative	
Acid-fast bacilli stain	Negative	
Cartridge-based nucleic acid amplification test	Negative	
AUTOIMMUNE ENCEPHALITIS PANEL	Negative	

Flaccid quadriplegia, though suggestive of both GBS and BBE, the presence of a low sensorium, an impaired oculocephalic reflex, and the presence of seizures favors the greater possibility of probable BBE. In view of poor clinical improvement and considering the differentials, we initiated the patient on intravenous immunoglobulins (IVIG) at a dose of 400 mg/kg once daily for five days at a daily dose of 30 grams per day. We noted a significant clinical response after day 2 of immunoglobulin therapy, including turning the head on command and improvement in limb power to 2/5. Truncal and limb ataxia and minimal lateral rectus palsy were noted during treatment, which resolved completely. Gradual improvement in sensorium, muscle power, and respiratory efforts was evident. Given clinical improvement and cost constraints, further investigations, including anti-GQ1b antibody testing, CSF oligoclonal bands, CSF IgG index, synthesis studies, and paraneoplastic evaluation, were not performed. A multidisciplinary approach involving a physiotherapist, clinical nutritionist, speech therapist, and respiratory therapist was undertaken. We gradually weaned the patient from mechanical ventilation to bi-level positive airway pressure and subsequently transferred him to the ward. In the ward, continuous bilevel positive airway pressure (BIPAP) support was weaned to intermittent support. Patient’s muscle power improved to 4+/5, and assisted ambulation was initiated. Oxygen requirements were down-titrated, and the patient tolerated continuous room-air ventilation. Fiber optic laryngoscopy showed normal vocal cord mobility. Gradual unassisted ambulation was attempted as a significant improvement in muscle power of 4+/5 in all four limbs was noted. By one week after initiation of IVIG, there was no evidence of sensory or cranial nerve dysfunction, complete resolution of ophthalmoplegia, and he was tolerating oral solid feeds and unaided ventilation post-decannulation of his tracheostomy tube. Regular periodic outpatient evaluation was performed, and a six-month review showed no residual neurological dysfunction, evidenced by peripheral power of 5/5 in all four limbs, no sensory dysfunction, and intact cranial nerve function.

## Discussion

BBE is a rare autoimmune neurological disorder within the spectrum of antiganglioside antibody-mediated diseases, closely related to GBS and Miller Fisher syndrome. It is classically characterized by the triad of ophthalmoplegia, ataxia, and altered consciousness, typically following an infectious trigger. However, considerable clinical heterogeneity exists, making early diagnosis challenging.

In contrast to classical descriptions, our patient presented with acute-onset altered sensorium and rapidly progressive quadriplegia. This required ventilatory support and occurred without a clear antecedent infection. Although we noted ophthalmoplegia and low sensorium, the absence of supportive findings on neuroimaging and CSF analysis posed a significant diagnostic dilemma. Notably, there was no evidence of albuminocytological dissociation in the CSF. MRI of the brain and spine was unremarkable. These findings are consistent with previous data indicating that only a subset of patients with BBE demonstrate radiological abnormalities and CSF findings. A significant fraction of patients may have normal imaging and CSF studies. Electrophysiological studies in our patient revealed severe sensorimotor neuropathy involving all four limbs. This further supports the diagnosis within the GBS spectrum. The overlap between BBE, GBS, and Miller Fisher syndrome is well recognized. All share immunopathogenesis mediated by antiganglioside antibodies, particularly anti-GQ1b. While GBS predominantly affects the peripheral nervous system and nerve roots, BBE demonstrates additional involvement of the brainstem reticular formation and central ocular pathways. Flaccid quadriplegia in this patient suggests both GBS and BBE. However, decreased sensorium with significantly low GCS, impaired oculocephalic reflex, and the presence of seizures favor probable BBE. Low sensorium in BBE is due to involvement of the reticular activating system within the brainstem. There was no characteristic descending paralysis as expected in GBS. In general, antibody testing may not always be possible in developing nations with limited medical facilities. In such scenarios, clinical judgment remains paramount.

Initiation of IVIG resulted in rapid and marked clinical improvement in sensorium, motor, and respiratory function. This favorable response to immunotherapy supports the diagnosis of probable BBE within the GBS spectrum. It also highlights the importance of early therapeutic intervention, even in diagnostically uncertain cases.

Several atypical presentations of BBE have been described in the literature. Case with isolated cranial nerve involvement, absence of altered sensorium, or unusual radiological findings [5,6]. A young child with an initial snake envenomation presentation was diagnosed later with BBE [7]. Compared to these reports, our case is notable for the complete absence of supportive radiological and CSF findings despite profound neurological impairment. This underscores the variability of the disease spectrum and reinforces the concept that BBE remains primarily a clinical diagnosis.

The overlap of brainstem and peripheral nervous system involvement in our patient supports the concept that BBE and GBS share a common immunological substrate rather than existing as an isolated neurological disorder. Overall, this case emphasizes that BBE should be considered in patients presenting with acute encephalopathy and neuromuscular weakness, particularly when routine investigations are inconclusive. In clinical practice, overlap among anti-ganglioside antibody-mediated neurological disorders is increasingly recognized. Early recognition and prompt initiation of immunotherapy can result in excellent neurological recovery.

## Conclusions

BBE is a rare immune-mediated neurological disorder characterized by the classical clinical triad of ataxia, altered sensorium, and ophthalmoplegia; however, wide clinical presentations have been documented. Features such as the absence of albuminocytological dissociation, normal MRI, or negative antibodies cannot rule out the condition with certainty. In patients presenting with acute brainstem dysfunction or unexplained encephalopathy, a high index of clinical suspicion of BBE is essential after ruling out infectious etiologies. There usually remains a diagnostic overlap between GBS and BBE; In this patient, the presence of decreased sensorium, impaired oculocephalic reflex, and the presence of epileptiform activity favors the greater possibility of probable BBE. This case highlights the importance of considering other variants within the anti-ganglioside antibody spectrum. Prompt clinical judgment remains the cornerstone in achieving favorable outcomes. Early recognition and timely initiation of immunotherapy can result in dramatic and complete neurological recovery, unlike many other diseases. However, the limitation in the study is that further autoantibodies, such as anti-GQ1b, CSF oligoclonal band, CSF IgG index, and paraneoplastic evaluation, could not be undertaken due to significant clinical improvement with the initiation of IVIG treatment. The absence of antibody testing is a significant drawback in this case; however, it emphasizes the importance of clinical judgment and looking out for subtle details on examination for the suspicion of BBE, especially in developing countries, where not all investigations are always possible.

## References

[1] Koga M (2013). A nationwide survey of patients with Bickerstaff brainstem encephalitis: diversity of underlying mechanism (Article in Japanese). Rinsho Shinkeigaku.

[2] Odaka M, Yuki N, Yamada M, Koga M, Takemi T, Hirata K, Kuwabara S (2003). Bickerstaff's brainstem encephalitis: clinical features of 62 cases and a subgroup associated with Guillain-Barré syndrome. Brain.

[3] Ito M, Kuwabara S, Odaka M, Misawa S, Koga M, Hirata K, Yuki N (2008). Bickerstaff's brainstem encephalitis and Fisher syndrome form a continuous spectrum: clinical analysis of 581 cases. J Neurol.

[4] Hussain AM, Flint NJ, Livsey SA, Wong R, Spiers P, Bukhari SS (2007). Bickerstaff's brainstem encephalitis related to Campylobacter jejuni gastroenteritis. J Clin Pathol.

[5] Cuneo GL, Grazzini I, Guadagni M, Venturini E, Bianchi A (2016). An atypical Bickerstaff's brainstem encephalitis with involvement of spinal cord. Neuroradiol J.

[6] Bagaria AK, Vyas A, Mathur V, Ranawat CS, Singh M (2021). Bickerstaff brainstem encephalitis with isolated acute bilateral ophthalmoplegia: an unusual presentation. Ann Indian Acad Neurol.

[7] Meghana BL, Jain MK, Patnaik S (2024). Bickerstaff brainstem encephalitis masquerading as snake bite: a case report. Indian J Neurol.

